# Supplementary material for: The antiadipogenic effect of the pentacyclic triterpenoid isoarborinol is mediated by LKB1-AMPK activation
Source: PLoS One. 2025 Sep 3;20(9):e0330860. doi: 10.1371/journal.pone.0330860 (PMC12407445; doi:10.1371/journal.pone.0330860)
Supplement: S1 Raw image — (PDF) [file pone.0330860.s001.pdf]

Raw Western Bloot images for Figure 5. Protein expression of C/EBPα and PPARγ in 3T3 cells at day 10 of differentiation.

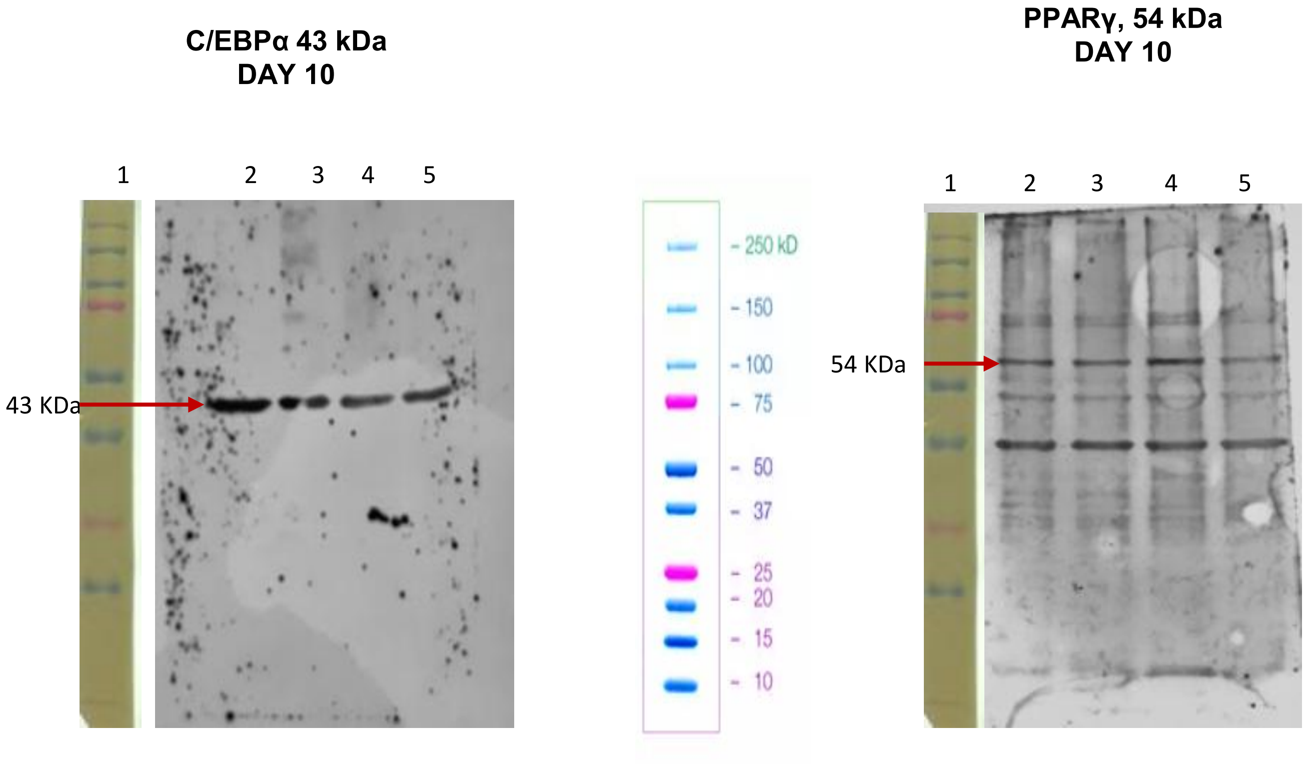

Representative Western blotting of C/EBPα and PPARγ.
1- Molecular weight (Precision Plus Protein Dual Color Standards, BioRad) 2- Control, 3- isoarborinol 0.36 µm, 4- isoarborinol 0.72 µm, 5- isoarborinol 1.44 µm.
Anti-CEBP Alpha/CEBPA antibody [EP709Y] (ab40764) (1:5000).
Anti-PPAR gamma antibody (ab209350), 1:5000.

Raw Western Bloot images for Figure 5. Protein expression of β Actin in 3T3 cells at day 10 of differentiation.

β-ACTIN, DAY 10

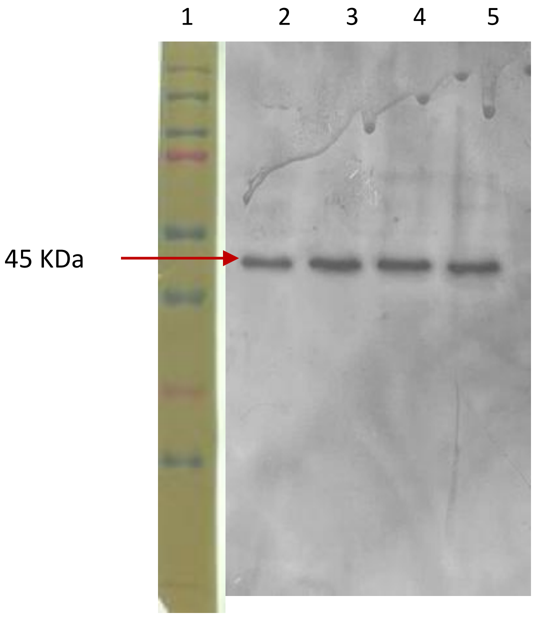

β-ACTIN, DAY 10

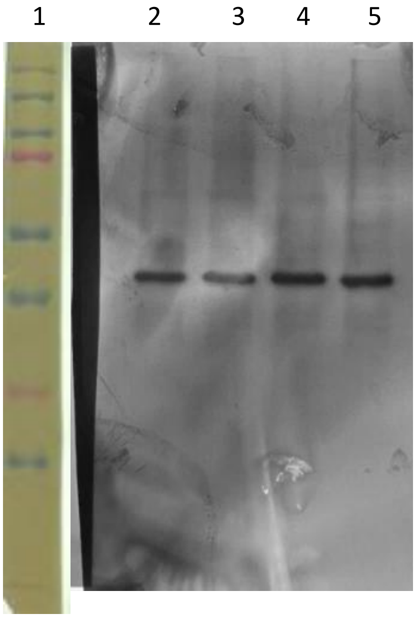

Western blot of β-Actin
1- Molecular weight (Precision Plus Protein Dual Color Standards, BioRad) 2- Control 3- isoarborinol 0.36 μm, 4- isoarborinol 0.72 μm, 5- isoarborinol 1.44 μm.
β-Actin (13E5) Rabbit mAb, 1:1000.

Raw Western Bloot images for Figure 6. Phosphorylation of LKB1 and AMPK in 3T3-L1 adipocytes treated with isoarborinol.

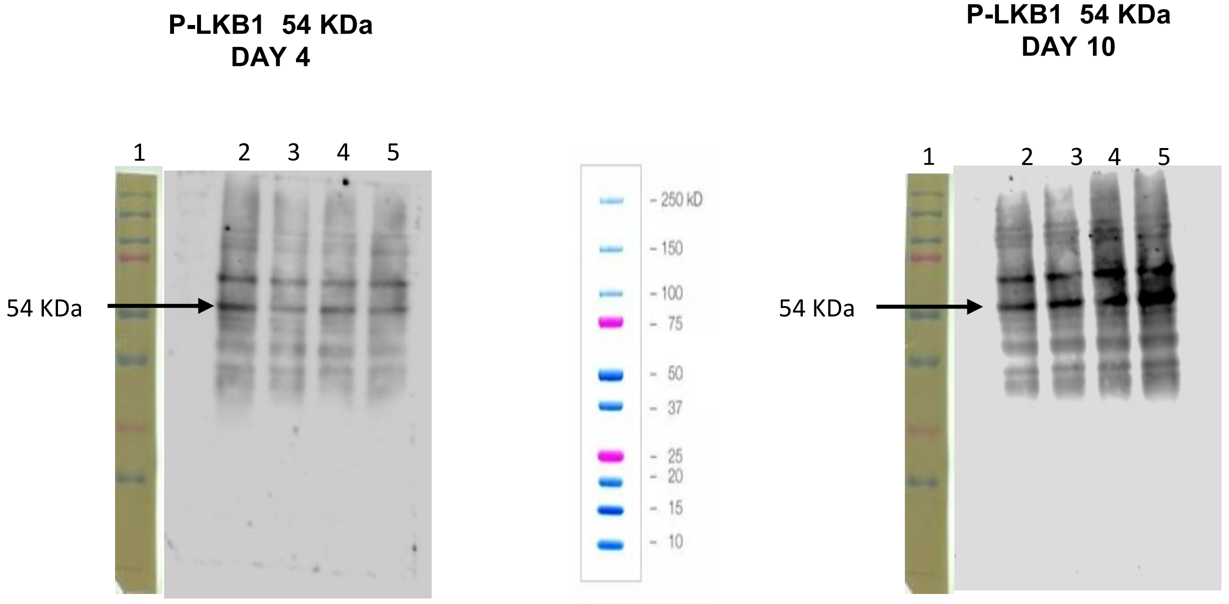

Western blot of p-LKB.
1- Molecular weight (Precision Plus Protein Dual Color Standards, BioRad) 2- isoarborinol 0 µm, 3- isoarborinol 0.36 µm, 4- isoarborinol 0.72 µm, 5- isoarborinol 1.44 µm.
Phospho-LKB1 (Ser428); (C67A3) Rabbit mAb, 1:5000.
Differentiating 3T3-L1 cells were treated with isoarborinol [0.36, 0.72 and 1.44 µM] and protein levels were determined at day 4 and 10 for western blotting assay

LKB1 54 KDa
DAY 4

LKB1 54 KDa
DAY 10

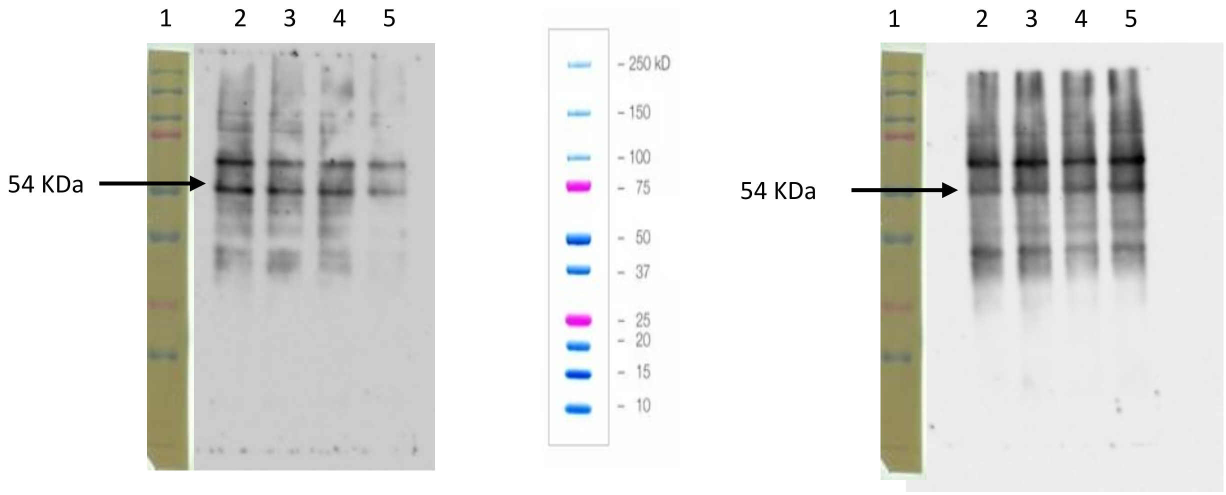

Western blot of p-LKB1.
1- Molecular weight (Precision Plus Protein Dual Color Standards, BioRad) 2- isoarborinol 0 μm, 3- isoarborinol 0.36 μm, 4- isoarborinol 0.72 μm, 5- isoarborinol 1.44 μm.
LKB1 (C60C5) Rabbit mAb, 1:5000.
Differentiating 3T3-L1 cells were treated with isoarborinol [0.36, 0.72 and 1.44 μM] and protein levels were determined at day 4 and 10 for western blotting assay

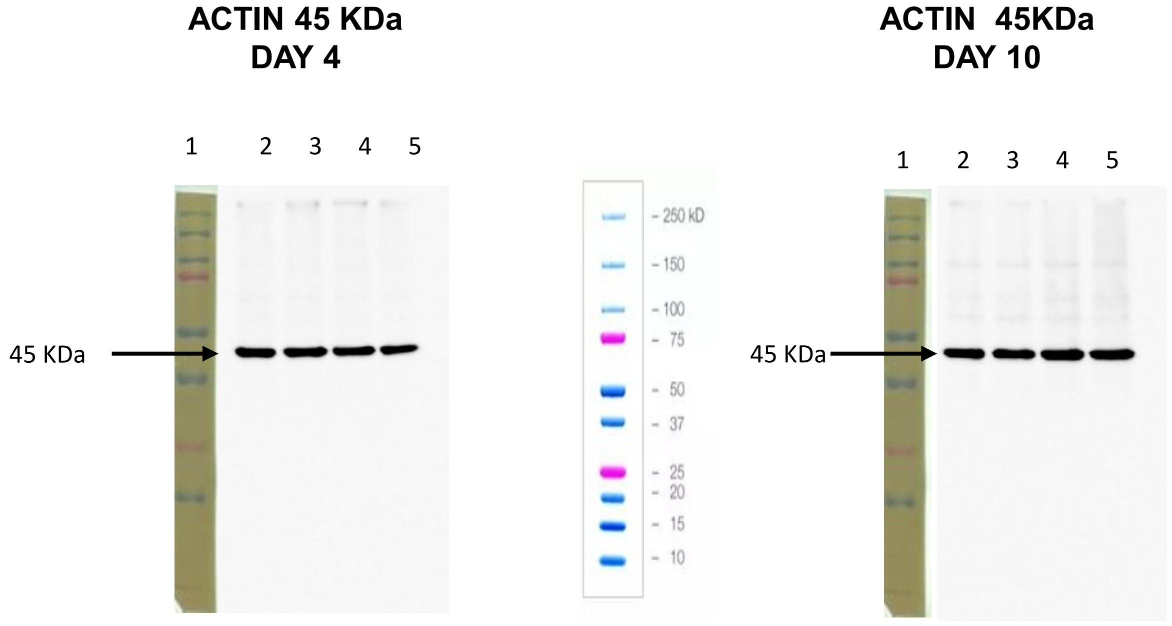

Western blot of β-Actin
1- Molecular weight (Precision Plus Protein Dual Color Standards, BioRad), 2- isoarborinol 0 μm, 3- isoarborinol 0.36 μm, 4- isoarborinol 0.72 μm, 5- isoarborinol 1.44 μm.
β-Actin (13E5) Rabbit mAb, 1:1000.
Differentiating 3T3-L1 cells were treated with isoarborinol [0.36, 0.72 and 1.44 μM] and protein levels were determined at day 4 and 10 for western blotting assay

B-Actin WB as a loading control.
Figure 6.

P-AMPK 62 KDa
DAY 4

P-AMPK 62 KDa
DAY 10

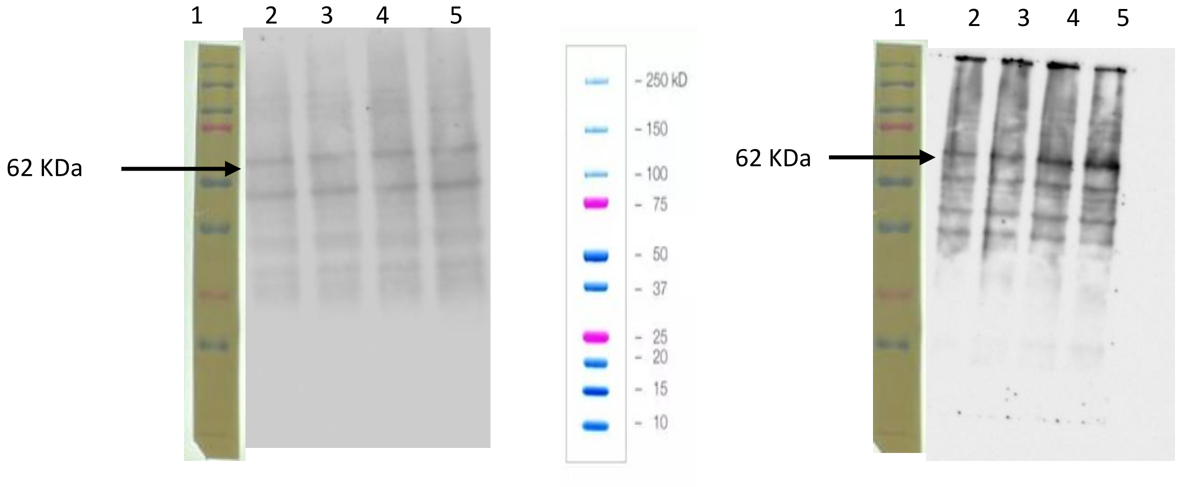

Western blot of P-AMPK
1- Molecular weight (Precision Plus Protein Dual Color Standards, BioRad) 2- isoarborinol 0 µm, 3- isoarborinol 0.36 µm, 4- isoarborinol 0.72 µm, 5- isoarborinol 1.44 µm.
Phospho-AMPKα (Thr172), (D4D6D), Rabbit mAb, 1:5000
Differentiating 3T3-L1 cells were treated with isoarborinol [0.36, 0.72 and 1.44 µM] and protein levels were determined at day 4 and 10 for western blotting assay

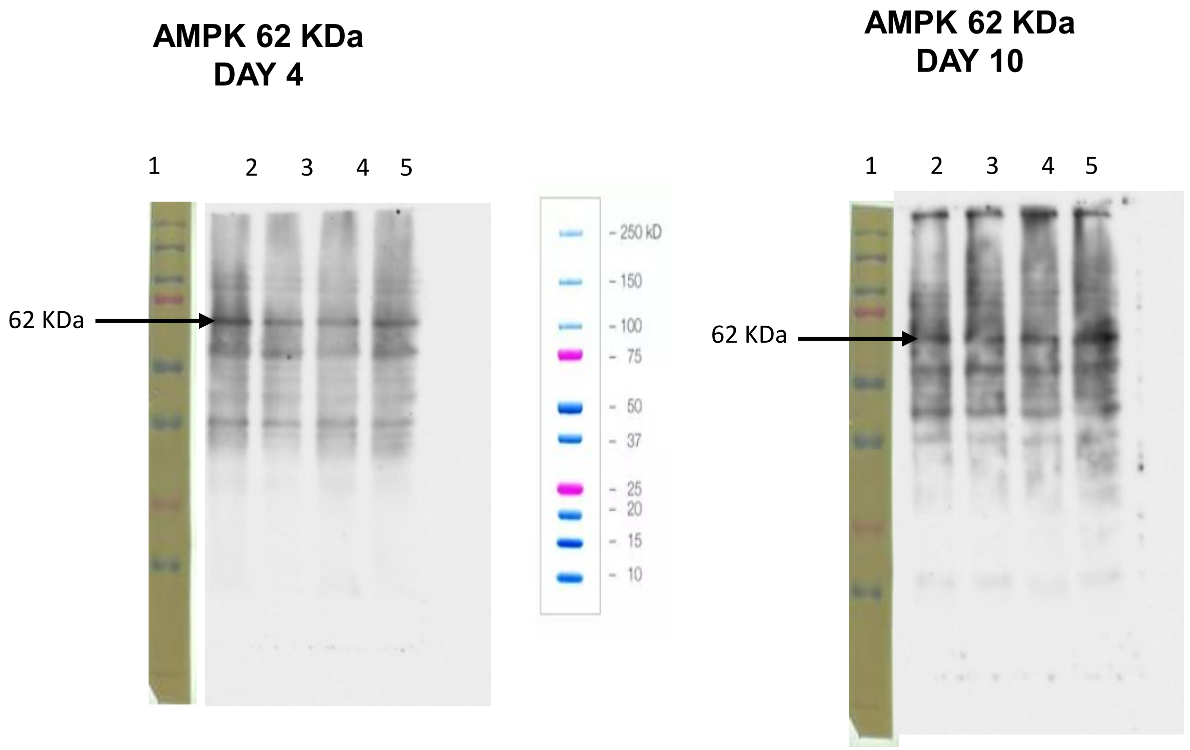

Western blot of AMPK

1- Molecular weight (Precision Plus Protein Dual Color Standards, BioRad) 2- isoarborinol 0 µm, 3- isoarborinol 0.36 µm, 4- isoarborinol 0.72 µm, 5- isoarborinol 1.44 µm.

AMPKα (D5A2), Rabbit mAb, 1:5000

Differentiating 3T3-L1 cells were treated with isoarborinol [0.36, 0.72 and 1.44 µM] and protein levels were determined at day 4 and 10 for western blotting assay

Values used to build graphs

Experimental data for Figure 1. Effect of isoarborinol on 3T3-L1 cell viability.

MTT24 h

			ISOARBORINOL CONCENTRATION [µm]					
	CONTROL	DMSO 1 %	0.36	0.72	1.44	2.88	5	50
	102	103.283086	101.621188	90.35313	75.5377207	71.667463	51.9582665	20.4494382
	100	96.1739908	92.8571429	94.7351525	78.8764045	73.1963688	58.635634	22.953451
	100	94.4302504	86.8057785	94.1091493	90.35313	76.8275203	50.2889246	24.8314607
	97	97.112928	93.6918138	100.160514	99.5345104	72.0496894	47.9935795	27.5441413
	100	93.2632585	93.692358	83.8844302	84.5104334	70.90301	39.2295345	16.9020867
	99	103.685488	82.2150883	78.6677368	89.3097913	70.5207836	50.2781	32.1348315
	99	113.074859	84.7191011	81.3804173	55.505618	65.7429527	47.38262	35.4735152
	98	98.0518651	96.8805504	83.8844302	85.7624398	74.7252747	51.6252	24.83678
	100	91.4793051	84.72	75.5377207	82.26233	71.667463	47.38262	26.6743413
		84.8542762		109.550562		70.1385571		
mean	99.4444444	97.5409307	90.8003357	89.2263243	82.4058198	71.7439083	49.4193866	25.7555607
sds	1.42400062	7.74605953	6.50605109	10.570302	12.2636755	2.93897663	5.14668672	5.64873647

MTT 48 h

			ISOARBORINOL CONCENTRATION [µm]					
	CONTROL	DMSO 1 %	0.36	0.72	1.44	2.88	5	50
	100	97.621226	86.7520586	78.7008234	79.3046661	71.667463	40.8600183	12.0768527
	100	94.6020128	81.9213175	95.4071363	91.802379	73.063688	34.6203111	16.7063129
	102.496153	88.3623056	92.9917658	83.3943275	69.6431839	76.8275203	31.6010979	20.5306496
	100	96.4135407	78.7008234	78.982617	94.994511	72.0496894	27.5754803	16.7063129
	100	89.36871	95.4071363	85.4071363	88.7648673	70.90301	29.78957	12.4123782
	102.120021	104.062214	93.3943275	86.533394	79.7072278	70.5207836	26.7703568	14.8572406
	97.9825611	100.237877	78.2982617	85.0059839	71.6559927	65.7429527	35.3564712	20.687297
	106.633613	97.0845746	95.0407136	84.8923919	78.6117285	67.654085	46.452385	26.7054197
	105.881347	97.2302958	82.0149188	85.1354071	79.921348	71.057463	33.990351	16.9954197
	101.679299	96.1091952	87.1690359	84.8288019	81.600656	71.0540728	34.1128935	17.5197648
	2.91568972	4.90667472	7.1341314	4.8613067	8.64063292	3.1387476	6.33696081	4.58084346

MTT 72 h

				ISOARBORINOL CONCENTRATION [µm]		
	Control	DMSO 1%	Differentiated	0.36	0.72	1.44
	105	96.1739908	99.1249361	83.1630046	96.5763924	83.9678079
	100	94.4302504	75.2491058	84.5043434	77.5293817	72.4322943
	100	97.112928	80.2120593	84.7726111	71.7616249	82.3582013
	100	92.2841083	95.7715892	97.3811957	79.0048544	86.1139499
	100	97.112928	94.832652	91.8817067	97.9177312	68.1400102
	98	103.685488	77.5293817	73.2370976	81.6875319	85.0408789
	99	113.074859	77.5293817	80.0779254	97.9177312	81.0168625
	99.998	98.0518651	97.2470618	81.0168625	81.6875319	93.759581
	99.99	91.0479305	76.3221768	74.8467041	79.1389882	86.5163516
			97.045861			
MEAN	100.220889	98.1082609	87.0864205	83.4312723	84.8024186	82.1495486
SD	1.92227004	6.69073293	10.3747181	7.61350086	9.9417045	7.69141459

Experimental data for Figure 3. Effect of isoarborinol on intracellular lipid accumulation in 3T3-L1 cells at day 10 of differentiation.

Lipid Acumulation (%)

		isoarborinol µM		
	Control	0.36	0.72	1.44
	100	82.3529412	80.8823529	75
	98.98272	95.890411	73.9726027	72.6027397
	100	97.2972973	81.0810811	68.9189189
	99.897	70.7142857	79.7142857	69.4285714
	99.7822	81.3333333	85.3333333	69.3333333
	97	73.9726027	76.7123288	76.7123288
	102.921	76.8363	81.083536	69.9231
	103	89.89756	76.8725262	73.96656
	100	83.742857	81.125622	71.9891
Mean	100.17588	83.559732	79.6419632	71.9860725
SDS	1.97527361	9.92447544	3.50924728	2.9991663

Experimental data for Figure 4. Effect of isoarborinol on the gene expression levels of transcription factors in 3T3-L1 preadipocytes (Day 4)

DAY 4

PPARγ

	isoarborinol μM		
CONTROL	0.36	0.72	1.44
1.0909	0.18685616	0.13678671	0.000054251
1.01	0.24827312	0.20026747	0.25173889
1.02	0.13030822	0.22845786	0.22531262
MEAN 1.0403	0.188479167	0.188504013	0.159035254
SDS 0.04410522	0.058999195	0.046954064	0.138314158

Technical Replicate Control

biological	a	b	c	mean
1	0.9091	1	1.3636	1.00909
2	0.99	1	1.02	1.01
3	1.01346	1.02346	1.03346	1.02346

Technical Replicate isoarborinol 0.36

biological	a	b	c	mean
1	0.17955	0.2	0.17856	0.18685616
2	0.194819	0.25	0.3	0.24827312
3	0.1209466	0.150001	0.12	0.13030822

Technical Replicate isoarborinol 0.72

biological	a	b	c	mean
1	0.13676671	0.14	0.13353342	0.13678671
2	0.20026747	0.21	0.19053494	0.20026747
3	0.32845786	0.12845786	0.22845786	0.22845786

Technical Replicate isoarborinol 1.44

biological	a	b	c	mean
1	0.000054251	0.0001	0.000008502	0.000054251
2	0.25173889	0.3	0.20347778	0.25173889
3	0.4	0.1	0.17593786	0.22531262

C/EBPα

	isoarborinol μM		
CONTROL	0.36	0.72	1.44
1.0367	0.71697762	0.22067575	0.17313868
1.005	0.85856544	0.28519093	0.18946457
1.03	0.75785828	0.30145196	0.15932008
1.0239	0.777800447	0.269106213	0.173974443
0.016707184	0.072870057	0.0427228	0.015089614

Technical Replicate isoarborinol control

biological	a	b	c	mean
1	1.5	1	0.6101	1.0367
2	1.2	0.9	0.915	1.005
3	1.03	1.1	0.96	1.03

Technical Replicate isoarborinol 0.36

biological	a	b	c	mean
1	0.71697762	0.9	0.53395524	0.71697762
2	1	0.85856544	0.71713088	0.85856544
3	0.75785828	0.8	0.71571656	0.75785828

Technical Replicate isoarborinol 0.72

biological	a	b	c	mean
1	0.257426	0.15653678	0.24806447	0.22067575
2	0.345676	0.22020779	0.289689	0.28519093
3	0.35	0.30145197	0.25290394	0.30145196

Technical Replicate isoarborinol 1.44

biological	a	b	c	mean
1	0.1673589	0.156742	0.19531444	0.17313868
2	0.2	0.18	0.18839371	0.18946457
3	0.145735	0.21475787	0.11746737	0.15932008

SREBP-1C

	isoarborinol μM		
CONTROL	0.36	0.72	1.44
1	0.34151006	0.29936968	0.36602142
1.0345	0.37631169	0.3685673	0.32759835
1.09892	0.23651441	0.29730178	0.26609255
1.04447333	0.31811205	0.32174625	0.31990411
0.05020849	0.07277651	0.0405614	0.0504068

Technical Replicate isoarborinol control

biological	a	b	c	mean
1	1.3	0.7	1	1
2	1.5	1	0.6035	1.0345
3	1.1	1.1	1.09676	1.09892

Technical Replicate isoarborinol 0.36

biological	a	b	c	mean
1	0.15789	0.2789996	0.58878058	0.34151006
2	0.27890978	0.4865479	0.36347739	0.37631169
3	0.1234567	0.25	0.2230288	0.23651441

Technical Replicate isoarborinol 0.72

biological	a	b	c	mean
1	0.345633	0.2422345	0.31024154	0.29936968
2	0.342168	0.456437	0.3070969	0.3685673
3	0.26756053	0.23468	0.38975	0.29730178

Technical Replicate isoarborinol 1.44

biological	a	b	c	mean
1	0.38938126	0.356343	0.35234	0.36602142
2	0.33592785	0.323424	0.3234432	0.32759835
3	0.256346	0.26849965	0.273432	0.26609255

C/EBPβ

	isoarborinol μM		
CONTROL	0.36	0.72	1.44
1	0.55478474	0.43226862	0.35355339
1.000886	0.74742462	0.39502066	0.4061262
1.0942937	0.58236679	0.57038186	0.52850902
1	0.62416527	0.40053494	0.4006
1.023794925	0.627185355	0.44955152	0.422197153
0.047001039	0.085082616	0.0822089	0.074696925

Technical Replicate Control

biological	a	b	c	mean
1	0.95	1	1.05	1
2	1.1	0.9	1.002658	1.000886
3	1.0828811	0.956	1.25	1.0942937

Technical Replicate isoarborinol 0.36

biological	a	b	c	mean
1	0.44467722	0.654377	0.5653	0.55478474
2	0.60896266	0.87688	0.7564312	0.74742462
3	0.54772837	0.684386	0.513986	0.58236679
	0.65054481	0.657843	0.564108	0.62416527

Technical Replicate isoarborinol 0.72

biological	a	b	c	mean
1	0.44985206	0.4561009	0.3908529	0.43226862
2	0.33776458	0.360765	0.4865324	0.39502066
3	0.53702371	0.6098	0.56432187	0.57038186
	0.4	0.4	0.40160482	0.40053494

Technical Replicate isoarborinol 1.44

biological	a	b	c	mean
1	0.50148327	0.3136879	0.245489	0.35355339
2	0.1715186	0.478964	0.567896	0.4061262
3	0.42137706	0.65428	0.50987	0.52850902
	0.22301	0.54679	0.432	0.40053494

C/EBPδ

	isoarborinol μM		
CONTROL	0.36	0.72	1.44
1.05	0.85263489	0.82359102	0.79291519
1.00012	0.90125046	0.87660572	0.70710678
1.00008	0.91272782	0.85681268	0.72698626
1.016733333	0.88887106	0.85233647	0.74233608
0.028809785	0.03190184	0.02678931	0.04491641

Technical Replicate Control

biological	a	b	c	mean
1	1.3764	0.876	0.8976	1.05
2	1.1	0.9	1.00036	1.00012
3	1.000024	1.1	0.9	1

Technical Replicate isoarborinol 0.36

biological	a	b	c	mean
1	0.67274467	0.89756	0.9876	0.85263489
2	1.1	0.8	0.80375138	0.90125046
3	0.83750696	1.0009	0.8997765	0.91272782

Technical Replicate isoarborinol 0.72

biological	a	b	c	mean
1	0.80788306	0.87646	0.78643	0.82359102
2	0.82981716	0.95	0.85	0.87660572
3	0.8	1	0.77043804	0.85681268

Technical Replicate isoarborinol 1.44

biological	a	b	c	mean
1	0.69248857	0.897657	0.7886	0.79291519
2	0.53703034	0.90887	0.67542	0.70710678
3	0.64522978	0.856754	0.678975	0.72698626

Experimental data for Figure 4. Effect of isoarborinol on the gene expression levels of transcription factors in 3T3-L1 preadipocytes (Day 10)

DAY 10

PPARγ

	isoarborinol μM		
CONTROL	0.36	0.72	1.44
1.01	0.87660572	0.29936968	0.09407792
1.022	0.52850902	0.4413515	0.02105052
0.9902	0.8122524	0.40053494	0.1907824
	0.62416527		
MEAN	1.0074	0.73912238	0.380418707
SDS	0.016058643	0.185212926	0.07309724

C/EBPα

	isoarborinol μM		
CONTROL	0.36	0.72	1.44
1	0.84089642	0.84674531	0.6328783
1	0.9862327	0.93952275	0.57434918
1.04	0.71315994	0.84674531	0.7120251
1.02	0.7845841	0.57038186	0.67830216
1.02	0.827992247	0.785549973	0.654892147
0.02	0.141617031	0.192028386	0.071761315

SREBP-1C

CONTROL	0.36	0.72	1.44
1.033	0.41465977	0.32759835	0.07432544
1.034	0.51763246	0.46976137	0.12940812
1.00022	0.44442134	0.35848881	0.11582351
1.02240667	0.45890452	0.38528284	0.10651902
0.01922072	0.05299213	0.07477313	0.02869591

PPARγ — Technical Replicate Control

biological	a	b	c	mean
1	0.81695	1.23765	0.9754	1.01
2	0.9696	1.1211	0.9753	1.022
3	0.98	1	0.9906	0.9902

C/EBPα — Technical Replicate Control

biological	a	b	c	mean
1	1.039	1.0637	0.8973	1
2	0.8445	0.9863	1.2892	1.04
3	1.0502	1.0228	0.987	1.02

SREBP-1C — Technical Replicate Control

biological	a	b	c	mean
1	0.885565	1.33678	0.876655	1.033
2	1.034	1.1	0.968	1.034
3	0.92636	1.0756	0.9987	1.00022

PPARγ — Technical Replicate 0.36

biological	a	b	c	mean
1	0.94574716	0.78654	0.89753	0.87660572
2	0.774892706	0.46876	0.342	0.52850902
3	0.8975809	0.6468976	0.8922787	0.8122524
	0.61802804	0.67546777	0.579	0.62416527

C/EBPα — Technical Replicate 0.36

biological	a	b	c	mean
1	0.67318926	0.9863	0.8632	0.84089642
2	1.1575249	0.8729	0.9282732	0.9862327
3	0.71467982	0.7823	0.6425	0.71315994
	0.7682	0.7133223	0.87223	0.7845841

SREBP-1C — Technical Replicate isoarborinol 0.36

biological	a	b	c	mean
1	0.45288731	0.37282	0.418272	0.41465977
2	0.56292	0.53714738	0.45283	0.51763246
3	0.4522	0.53814402	0.34292	0.44442134

PPARγ — Technical Replicate isoarborinol 0.72

biological	a	b	c	mean
1	0.38434334	0.138965	0.3748007	0.29936968
2	0.2361345	0.43562	0.6523	0.4413515
3	0.63736	0.22200392	0.3422409	0.40053494

C/EBPα — Technical Replicate 0.72

biological	a	b	c	mean
1	0.87996693	0.876639	0.78363	0.84674531
2	0.98726	1.00428825	0.82702	0.93952275
3	0.78932	0.76383	0.98708593	0.84674531
	0.44032558	0.6982	0.57262	0.57038186

SREBP-1C — Technical Replicate isoarborinol 0.72

biological	a	b	c	mean
1	0.38331305	0.326282	0.2732	0.32759835
2	0.5622	0.39088411	0.4562	0.46976137
3	0.48493723	0.2633	0.3272292	0.35848881

PPARγ — Technical Replicate isoarborinol 1.44

biological	a	b	c	mean
1	0.145419318	0.06788922	0.068925222	0.09407792
2	0.01419656	0.012233	0.036722	0.02105052
3	0.2448242	0.218293	0.10923	0.1907824

C/EBPα — Technical Replicate 1.44

biological	a	b	c	mean
1	0.5735149	0.5679	0.75722	0.6328783
2	0.56927	0.48085644	0.6729211	0.57434918
3	0.89732	0.7234312	0.5153241	0.7120251
	0.6752	0.76248648	0.59722	0.67830216

SREBP-1C — Technical Replicate isoarborinol 1.44

biological	a	b	c	mean
1	0.08757632	0.0632	0.0722	0.07432544
2	0.1223	0.16731236	0.098612	0.12940812
3	0.12634191	0.122366	0.09876262	0.11582351

Experimental data for Figure 5. Protein expression of C/EBPα and PPARγ in 3T3 cells at day 10 of differentiation.

DAY 10

PPARγ/actin

Control	0.36	0.72	1.44
1.58120526	0.88162126	1.15998855	0.81048395
1.64737451	0.92674238	1.21935644	0.85196428
1.49153073	0.91578811	1.20494342	0.84189391
1.57337017	0.90805058	1.1947628	0.83478071
0.07821677	0.02353467	0.03096563	0.02163568

CEBPα/actin

Control	0.36	0.72	1.44
0.93178298	0.58871963	0.47822804	0.347675
1.13867187	0.61885013	0.50270361	0.36546891
1.18280061	0.6115352	0.49676156	0.361149
1.08441849	0.60636832	0.4925644	0.35809764
0.13401505	0.01571573	0.01276618	0.00928111

PPARγ/actin — Technical Replicate Control

biological	a	b	c	mean
1	1.77281578	1.2974	1.6734	1.58120526
2	1.4593	1.80459353	1.67823	1.64737451
3	1.3834	1.5034	1.58779219	1.49153073

CEBPα/actin — Technical Replicate Control

biological	a	b	c	mean
1	0.93926894	0.96374	0.89234	0.93178298
2	1.2934	1.1834344	0.93918121	1.13867187
3	1.19744	1.36344	0.98752183	1.18280061

PPARγ/actin — Technical Replicate 0.36

biological	a	b	c	mean
1	0.8634	0.7823	0.99916378	0.88162126
2	0.9234	0.92848714	0.92834	0.92674238
3	0.98234	0.87158433	0.89344	0.91578811

CEBPα/actin — Technical Replicate 0.36

biological	a	b	c	mean
1	0.53931889	0.6344	0.59244	0.58871963
2	0.61728344	0.67633295	0.562934	0.61885013
3	0.6234543	0.6918073	0.519344	0.6115352

PPARγ/actin — Technical Replicate0.72

biological	a	b	c	mean
1	1.1574	1.11282565	1.20974	1.15998855
2	1.3723	0.99642932	1.28934	1.21935644
3	1.12292626	1.29834	1.193564	1.20494342

CEBPα/actin — Technical Replicate0.72

biological	a	b	c	mean
1	0.47934	0.56588912	0.389455	0.47822804
2	0.562934	0.458343	0.48683383	0.50270361
3	0.497343	0.59449168	0.39845	0.49676156

PPARγ/actin — Technical Replicate 1.44

biological	a	b	c	mean
1	0.81023	0.83792185	0.7833	0.81048395
2	0.87293	0.89366284	0.7893	0.85196428
3	0.8603834	0.7129343	0.95236403	0.84189391

CEBPα/actin — Technical Replicate 1.44

biological	a	b	c	mean
1	0.389135	0.35645	0.29744	0.347675
2	0.32734	0.36733673	0.40173	0.36546891
3	0.37495	0.410042	0.298455	0.361149

Experimental data for Figure 6. Phosphorylation of LKB1 and AMPK in 3T3-L1 adipocytes treated with isoarborinol.

Day 4

LKB1/pLKB1

	Control	0.36	0.72	1.44
	0.76972278	0.45356799	0.57439839	0.1678
	0.673	0.45721	0.5673	0.276
	0.65376153	0.526	0.46989	0.17574
mean	0.6988281	0.478926	0.53719613	0.20651333
SDS	0.06214556	0.04080793	0.05839677	0.06030803

AMPK/pAMPK

	Control	0.36	0.72	1.44
	0.22408519	0.15711529	0.134	0.34848923
	0.235	0.1563	0.178	0.356
	0.23153	0.14733	0.202	0.4565
mean	0.23020506	0.15358176	0.17133333	0.38699641
SDS	0.00557673	0.00542951	0.03448671	0.06030891

LKB1/pLKB1 — Technical Replicate Control

biological	a	b	c	mean
1	0.84773434	0.789234	0.6722	0.76972278
2	0.7845	0.6723	0.5622	0.673
3	0.673	0.72	0.56828459	0.65376153

AMPK/pAMPK — Technical Replicate Control

biological	a	b	c	mean
1	0.24595557	0.234	0.1923	0.22408519
2	0.19022	0.23648	0.2783	0.235
3	0.23391	0.29723	0.16345	0.23153

LKB1/pLKB1 — Technical Replicate 0.36

biological	a	b	c	mean
1	0.45723	0.53524397	0.36823	0.45356799
2	0.486618	0.456	0.429012	0.45721
3	0.622	0.452	0.504	0.526

AMPK/pAMPK — Technical Replicate 0.36

biological	a	b	c	mean
1	0.16723	0.16981587	0.1343	0.15711529
2	0.1672	0.1283	0.1734	0.1563
3	0.1435	0.16352	0.13497	0.14733

LKB1/pLKB1 — Technical Replicate0.72

biological	a	b	c	mean
1	0.65809517	0.5664	0.4987	0.57439839
2	0.6352	0.4533	0.6134	0.5673
3	0.47795	0.522	0.40972	0.46989

AMPK/pAMPK — Technical Replicate0.72

biological	a	b	c	mean
1	0.234	0.139	0.029	0.134
2	0.143	0.218	0.173	0.178
3	0.19836	0.2093	0.19834	0.202

LKB1/pLKB1 — Technical Replicate 1.44

biological	a	b	c	mean
1	0.1569	0.1642	0.1823	0.1678
2	0.18922	0.2783	0.36048	0.276
3	0.14523	0.20679	0.1752	0.17574

AMPK/pAMPK — Technical Replicate 1.44

biological	a	b	c	mean
1	0.3723	0.37456769	0.2986	0.34848923
2	0.37347	0.2973	0.39723	0.356
3	0.42733	0.53194	0.41023	0.4565

DAY 10

LKB1/pLKB1

	Control	0.36	0.72	1.44
	1.27072051	1.14195346	1.34293151	2.52693324
	1.2896554	1.134554	1.3656	2.5
	1.233434	1.234213	1.34456	2.65464
mean	1.2646033	1.17024015	1.3510305	2.56052441
SDS	0.02860553	0.05552551	0.0126438	0.08261149

AMPK/pAMPK

	Control	0.36	0.72	1.44
	1.199	1.1373413	1.3232	1.4184168
	1.232423	1.12313	1.3392289	1.55554
	1.2324764	1.09292	1.4344	1.5431
mean	1.2212998	1.1177971	1.36560963	1.5056856
SDS	0.01931221	0.02268574	0.06011088	0.07583252

LKB1/pLKB1 — Technical Replicate Control

biological	a	b	c	mean
1	1.41609853	1.212833	1.18323	1.27072051
2	1.21604347	1.27383	1.37909273	1.2896554
3	1.328217	1.137465	1.23462	1.233434

AMPK/pAMPK — Technical Replicate Control

biological	a	b	c	mean
1	1.18373	1.28697	1.1263	1.199
2	1.19823	1.260319	1.23872	1.232423
3	1.17653	1.2505592	1.27034	1.2324764

LKB1/pLKB1 — Technical Replicate 0.36

biological	a	b	c	mean
1	1.23480038	1.19833	0.99273	1.14195346
2	1.1273943	1.1384744	1.1377933	1.134554
3	1.163793	1.267312	1.271534	1.234213

AMPK/pAMPK — Technical Replicate 0.36

biological	a	b	c	mean
1	1.127849	1.14445046	1.13972444	1.1373413
2	1.1190983	0.9528517	1.29744	1.12313
3	1.092983	1.07833	1.107447	1.09292

LKB1/pLKB1 — Technical Replicate 0.72

biological	a	b	c	mean
1	1.31717053	1.268394	1.44323	1.34293151
2	1.376253	1.290714	1.429833	1.3656
3	1.3372563	1.29834	1.3980837	1.34456

AMPK/pAMPK — Technical Replicate 0.72

biological	a	b	c	mean
1	1.3374574	1.2581986	1.373944	1.3232
2	1.4181807	1.309873	1.289633	1.3392289
3	1.47023	1.26967	1.5633	1.4344

LKB1/pLKB1 — Technical Replicate 1.44

biological	a	b	c	mean
1	2.527394	2.54418572	2.50922	2.52693324
2	2.65384	2.33596	2.5102	2.5
3	2.6782	2.6884	2.59732	2.65464

AMPK/pAMPK — Technical Replicate 1.44

biological	a	b	c	mean
1	1.56	1.545972	1.1492784	1.4184168
2	1.568903	1.551497	1.54622	1.55554
3	1.52873	1.704848	1.395722	1.5431

Experimental data for Figure 7. Effect of isoarborinol on the gene expression levels of ACC1, FAS, and FABP4 in differentiating 3T3-L1 adipocytes.

DAY 10

ACC

	CONTROL	isoarborinol µM 0.36	0.72	1.44
	1.012	0.52485834	0.54336743	0.32533546
	1.03	0.68460006	0.46759562	0.39502066
	1.03107	0.79553648	0.61132014	0.3519234
MEAN	1.02435667	0.668331627	0.540761063	0.357426507
SD	0.01071455	0.136070424	0.0718977	0.035167029

FABP4

	CONTROL	isoarborinol µM 0.36	0.72	1.44
	1.0357	0.89089872	0.35437122	0.4665165
	1.04	0.85819742	0.22635619	0.41370281
	1.023	0.82549612	0.4774208	0.41871281
MEAN	1.0329	0.85819742	0.35271607	0.43297737
SD	0.00883912	0.0327013	0.12554049	0.02915356

FAS

	CONTROL	isoarborinol µM 0.36	0.72	1.44
	1.0129	0.93952275	0.38024469	0.42337266
	1.024	0.87660572	0.24913507	0.39502066
	1.01134	0.8849342	0.3103789	0.3978086
MEAN	1.01608	0.90035422	0.31325289	0.40540064
SD	0.00690313	0.03417559	0.06560204	0.01562652

ACC — Technical Replicate Control

biological	a	b	c	mean
1	1.0419	1.022	0.9721	1.012
2	1.0893	0.873	1.1277	1.03
3	1.016565	0.9932	1.083445	1.03107

FABP4 — Technical Replicate Control

biological	a	b	c	mean
1	1.2427	0.9722	0.8922	1.0357
2	1.0034	1.1244	0.9922	1.04
3	0.973	1.103659	0.992341	1.023

FAS — Technical Replicate Control

biological	a	b	c	mean
1	1.01946	1.0332	0.98604	1.0129
2	1.0453	1.043277	0.983423	1.024
3	1.024	1.00657	1.00345	1.01134

ACC — Technical Replicate isoarborinol 0.36

biological	a	b	c	mean
1	0.5633	0.37914102	0.632134	0.52485834
2	0.690233	0.79964598	0.5639212	0.68460006
3	0.679332	0.86895744	0.83832	0.79553648

FABP4 — Technical Replicate isoarborinol 0.36

biological	a	b	c	mean
1	0.97363	0.82783216	0.871234	0.89089872
2	0.85373	0.85352226	0.86734	0.85819742
3	0.83425	0.82500436	0.817234	0.82549612

FAS — Technical Replicate isoarborinol 0.36

biological	a	b	c	mean
1	0.92234	0.96388825	0.93234	0.93952275
2	0.8752393	0.88923286	0.865345	0.87660572
3	0.8762034	0.8892342	0.889365	0.8849342

ACC — Technical Replicate isoarborinol 0.72

biological	a	b	c	mean
1	0.49965029	0.457222	0.67323	0.54336743
2	0.46134386	0.469223	0.47222	0.46759562
3	0.68436333	0.628365	0.52123209	0.61132014

FABP4 — Technical Replicate isoarborinol 0.72

biological	a	b	c	mean
1	0.41344166	0.356232	0.29344	0.35437122
2	0.212442	0.23319657	0.23343	0.22635619
3	0.5554004	0.394532	0.48233	0.4774208

FAS — Technical Replicate isoarborinol 0.72

biological	a	b	c	mean
1	0.389023	0.36247707	0.389234	0.38024469
2	0.23493521	0.24698	0.26549	0.24913507
3	0.319834	0.3100627	0.30124	0.3103789

ACC — Technical Replicate isoarborinol 1.44

biological	a	b	c	mean
1	0.32876198	0.3189044	0.32834	0.32533546
2	0.389024	0.40102058	0.3950174	0.39502066
3	0.346295	0.3519352	0.35754	0.3519234

FABP4 — Technical Replicate isoarborinol 1.44

biological	a	b	c	mean
1	0.43782	0.5088175	0.452912	0.4665165
2	0.4192033	0.36367113	0.458234	0.41370281
3	0.418923	0.41787543	0.41934	0.41871281

FAS — Technical Replicate isoarborinol 1.44

biological	a	b	c	mean
1	0.428394	0.42928398	0.41244	0.42337266
2	0.370236	0.40448098	0.410345	0.39502066
3	0.379023	0.401234	0.4131688	0.3978086
