# Supplementary material for: The antiadipogenic effect of the pentacyclic triterpenoid isoarborinol is mediated by LKB1-AMPK activation
Source: PLoS One. 2025 Sep 3;20(9):e0330860. doi: 10.1371/journal.pone.0330860 (PMC12407445; doi:10.1371/journal.pone.0330860)
Supplement: Attachment — Submitted filename: ReviewComments_PlosOne_01.10.25.docx [file pone.0330860.s002.docx]

In this manuscript titled, “The pentacyclic triterpenoid isoarborinol inhibits adipogenesis through c/EBPβ, PPARγ and SREBP-1C downregulation and LKB1-AMPK phosphorylation”, the authors Arcos-Reyes et al, show the safety and efficacy of isoarborinol to inhibit adipogenesis in 3T3-L1 cell system. The manuscript would be perfect for publication in PLoS One journal after addressing the following major comments:

1. Throughout the manuscript the authors compared increasing concentrations of isoarborinol with no isoarborinol treated cells. The ideal control to use in all experiments would be the vehicle i.e. DMSO since the compound was dissolved in DMSO. Care needs to be taken to make sure the increasing concentrations of isoarborinol donot increase DMSO ruling out any effects of the vehicle. In line with this, the authors would then change all control bars labeled as “0” in Isoarborinol concentration as DMSO or Vehicle for clarity of the readers.
2. While the toxicity assay is performed in 3T3-L1 fibroblasts in Figure 1, further experiments have been performed in differentiating preadipocytes. Hence, the toxicity of isoarborinol should be tested in differentiating preadipocytes. Alongwith the cell toxicity information, the cell proliferation effects of isoarborinol should also be checked.
3. The authors show, through Oil Red staining, the reduction in lipogenesis. The authors can enhance this point by assaying Triglycerides in the isoarborinol treated cells. This will support their claim. Moreover, it would be interesting to check changes in lipid accumulation on treatment with isoarborinol after differentiation of cells to adipocytes. This would answer the question, does the compound inhibits adipogenesis or lipid accumulation.
4. The authors show the significant reduction in key transcription factors of C/EBP, PPARgamma and SREBP-1C through quantitative PCR assay. Since RNA levels donot necessarily reflect protein levels, the RNA quantification should be accompanied by western blotting to check the protein levels in the isoarborinol treated condition. Additionally, the authors should show the variation of control in their qPCR data as the control would vary across replicates. This can be done by using the mean expression value of the controls, across replicates, as a reference to compare individual controls as well as the three isoarborinol concentration.
5. The authors show increased AMPK phosphorylation on isoarborinol treatment while the LKB1 shows increased phosphorylation towards day 10. The authors need to provide an explaination for the opposing LKB1 phosphorylation status at day 4 and day 10 of adipogenesis. Moreover, the phosphorylated proteins (LKB1 and AMPK) should also be compared with respective total proteins to get the extent of phosphorylation (or percentage of phosphorylation) which makes more sense than amount of phosphorylation since the protein itself can also change in amount.
6. The authors claim that isoarborinol inhibits adipogenesis through LKB1-AMPK pathway but only provide the amount of proteins as evidence. To make sure LKB1-AMPK pathway is indeed involved in isoarborinol effects, their components should be reduced with isoarborinol treatment to check for rescue of the effect observed. This is crucial to show LKB1-AMPK involved in isoarborinol effects as the protein levels can change as a consequence of the treatment rather than cause the effect.

Some minor comments are:

1. In the introduction paragraph 5, from lines 60-67 no reference has been provided for the claims made. Proper referencing needs to be done.
2. In figure 3 and figure 4, the y-axis of the graphs provides little information due to very few ticks provided. The graph should be corrected with increased ticks allowing the reader to infer the levels shown in the graph for each bar.
