# Supplementary material for: The antiadipogenic effect of the pentacyclic triterpenoid isoarborinol is mediated by LKB1-AMPK activation
Source: PLoS One. 2025 Sep 3;20(9):e0330860. doi: 10.1371/journal.pone.0330860 (PMC12407445; doi:10.1371/journal.pone.0330860)
Supplement: Attachment — Submitted filename: Response to Reviewer.docx [file pone.0330860.s005.docx]

**ANSWER TO REVIEWERS COMMENTS**

Reviewer #1: The authors have cited multiple studies where only qPCR levels of the genes such as C/EBP, PPARgamma and SREBP-1C have been provided. Here are multiple citations showing poor correlation between RNA levels and proteins (PMID: 11340206;PMID: 12952525; PMID: 27104977). Please provide western blots for the proteins. Multiple reviewers have asked the authors to perform treatment on differentiated cells and check lipid accumulation. Experiment needs to be performed.

**REPLY:** We thank the reviewer for his comments. We agree with the reviewer about the fact that RNA levels do not necessarily reflect protein levels.

We performed a Western blotting assay to evaluate the effect of isoarborinol on protein expression of C/EBPα and PPARγ 10 days after adipocyte differentiation. The results show that isoarborinol not only affects mRNA expression of C/EBPα and PPARγ, but also their protein expression, which supports that the anti-adipogenic effect of isoarborinol is associated with down regulation in the expression of these master regulators of adipogenesis, and associated pathways.

We have included Figure 5, which shows these results.

We have included the antibody data used in *Materials and Methods*, lines 177-178.

The caption for Figure 5 has been included in the *Results* section. Lines 285-294.

Some comments have been included in the *Discussion* section. Lines 356, 357, 429, and 430.

In reference to perform treatment on differentiated cells and check lipid accumulation.

**REPLY**: In this new version of the work, we have evaluated the changes in the expression of lipogenic genes in adipocytes treated with isoarborinol and it was found that cells treated with isoarborinol had decreased expression of ACC1, FAS and FAB1 (Fig 7), which corresponds to the decrease in intracellular lipids observed by Oil Red O staining (Fig 3). We have included this in the document and discussed these findings with those published by other authors. Page 20, lines 402-409. These experiments are consistent with our goal of evaluating the effect of isoarborinol on adipogenesis.

We understand that treating differentiated cells with isoarborinol and check lipid accumulation is a very interesting question, but this was not the goal of this study. As we explained before, this is the first study that evaluate the potential of isoarborinol for obesity treatment, and as many reports do, we decided to evaluate its effects on adipogenesis, including a possible mechanism of action. In future work, we will describe the effects of isoarborinol on differentiated adipocytes, including gene expression, lipid accumulation and molecular mechanism.
